# “*I didn’t realise they had such a key role*.” Impact of medical education curriculum change on medical student interactions with nurses: a qualitative exploratory study of student perceptions

**DOI:** 10.1007/s10459-019-09906-4

**Published:** 2019-08-07

**Authors:** Ray Samuriwo, Elinor Laws, Katie Webb, Alison Bullock

**Affiliations:** 1 School of Healthcare Sciences, Cardiff University, Ty Dewi Sant, Heath Park Campus, Cardiff, CF14 4XN UK; 2 Wales Centre for Evidence Based Care, Cardiff University, Cardiff, UK; 3 School of Medicine, Cardiff University, Cardiff, UK; 4 Centre for Medical Education, School of Medicine, Cardiff University, Cardiff, UK; 5 Cardiff Unit for Research and Evaluation in Medical and Dental Education (CUREMeDE), School of Social Sciences, Cardiff University, Cardiff, UK

**Keywords:** Medical students, Nurses, Medical education, Curriculum, Professional stereotypes, Interprofessional teamwork

## Abstract

Interprofessional teamwork between healthcare professionals is integral to the delivery of safe high-quality patient care in all settings. Recent reforms of medical education curricula incorporate specific educational opportunities that aim to foster successful interprofessional collaboration and teamwork. The aim of this study was to explore the impact of curriculum reform on medical students’ perceptions of their interactions and team-working with nurses. We gathered data from 12 semi-structured individual narrative interviews with a purposive sample of male (n = 6) and female (n = 6) medical students from fourth year (n = 6 following an integrated curriculum) and fifth year (n = 6 following a traditional curriculum). Data were subject to narrative analysis which was undertaken using NVivo software. Overall, there was no notable difference in the responses of the participants on the traditional and integrated curricula about their interactions and team work with nurses. However, the introduction of an integrated medical curriculum was viewed positively but a lack of interprofessional education with nursing students, removal of a nursing placement and shorter clinical placements were perceived as lost opportunities for the development of educationally beneficial relationships. The participants reported that nurses play a number of roles in clinical practice which underpin patient safety including being medical educators who provide a valuable source of support for medical students. The participants highlighted different factors that could hinder or foster effective working relationships such as a lack of understanding of nurses’ different professional roles and mutual respect. Medical education needs to provide students with more structured opportunities to work with and learn from nurses in clinical practice. Further research could explore how to foster positive relationships between medical students and nurses.

## Introduction

Interprofessional teamwork between healthcare professionals is integral to improving population health and the sustainable development of nations (United Nations 2015; WHO 2015). The delivery of safe, high quality healthcare is dependent on successful interprofessional communication and teamworking (Auerbach et al. 2012; Leonard et al. 2004; Welp and Manser 2016). Poor communication and teamwork between healthcare professionals are often identified as one of the main contributory factors to patient harm and adverse patient safety events (Thomas et al. 2010; Lingard et al. 2004; Brock et al. 2013). Effective interprofessional collaboration between different healthcare professionals has been found to prevent adverse patient safety events in healthcare are prevented in different contexts (Manser 2009; Sacks et al. 2015).

Doctors and nurses play a critical role in delivering healthcare, and unsurprisingly the nature of their relationship has a powerful influence on the quality of patient care (Tang et al. 2013; Siedlecki and Hixson 2015). Gender and perceived authority have historically been central in the relationship between doctors and nurses (Sweet and Norman 1995) but the adoption of a more pluralistic interprofessional approach to healthcare has altered this dynamic. The role of nurses has evolved to incorporate some aspects of medical work with a positive impact on patient outcomes but, this has contributed to conflict with doctors over jurisdiction in relation to patient care (Williamson et al. 2013; Ford 2015; Allen 2015; Salhani and Coulter 2009). Conflict between doctors and nurses can undermine interprofessional teamworking and consequently diminish the quality of patient care (Powell and Davies 2012; Dixon-Woods 2010). Stereotypes and attitudes relating to professional boundaries and gender are also known to exacerbate conflict between doctors and nurses (Braithwaite et al. 2013; Powell and Davies 2012).

Education is vital to the formation of highly skilled, dynamic and resilient healthcare professionals who deliver safe high-quality care (Cresswell et al. 2013; Robert et al. 2014). Some have argued that medical educators have not always been cognisant of education regarding interprofessional teamworking (Cooke et al. 2006). In some settings, interprofessional education has historically been optional rather than mandatory (Hammick et al. 2007; Ateah et al. 2011; Reeves et al. 2016). There have been some reports (Westbrook et al. 2008; Milne et al. 2015) that junior doctors often have a heavy clinical workload in clinical practice which limits their ability to work collaboratively with other healthcare professionals such as nurses. More recently, there has been a been a greater emphasis on the development and implementation of curricula that foster interprofessional teamworking which facilitates the consistent delivery of safe high-quality patient care (WHO 2010, 2011; GMC 2015, 2016). Interprofessional team-working can be fostered by educational curricula that foster meaningful social relationships in which students and educators from different professions learn from with and about each other (Ateah et al. 2011; Hean et al. 2012 Reeves et al. 2016). Effective team-working is subject to a number of factors such as the individual’s knowledge, attitude and perception of their own discipline as well as their views towards people from other professions (Ateah et al. 2011; Reeves et al. 2016). Therefore, curricula designed to foster interprofessional team-working must take into account the specific learning needs of students and enable them to understand the role of other healthcare professionals in the high-quality patient centred care (Hammick et al. 2007; Ateah et al. 2011; Reeves et al. 2016).

Perceptions and professional stereotypes have an impact on interprofessional collaboration and the quality of care (GMC 2016; Hall et al. 2016). There is a global consensus (WHO 2010, 2011) that IPE can help students to overcome hierarchical attitudes and professional stereotypes which in turn fosters effective teamwork. Medical students and junior doctors are expected to be aware of how to access support and work collaboratively with other healthcare professionals to ensure patient safety (GMC 2015, 2016). The involvement of nurse tutors in the medical education helps to challenge stereotypical views about other healthcare professions and provides useful learning opportunities (Walsh et al. 2014). One unpublished study (Samuriwo et al. 2016), reported that nurses supported medical students to develop their cognitive, functional, behavioural and ethical competence in medical practice to ensure patient safety during the transition to becoming junior doctors. Other studies (Jakobsen 2016; Visser et al. 2019) suggest that when medical students are on a clinical rotation in which they do not interact meaningfully with other healthcare professionals or get a thorough insight into the role of a nurse and nurses’ clinical decision-making processes. Arguably, a lack of a genuine insight into how nurses work and make decisions about patient care may contribute to conflict over jurisdiction which has a deleterious effect on interprofessional team-working. Therefore, it is important to establish the impact of fewer structured opportunities for clinical interprofessional teamwork on medical students’ perceptions of and team-working with nurses.

## Aim

Our aim in this study was to explore the impact of curriculum reform on medical students’ perceptions of their interactions and teamworking with nurses.

## Methods

### Theoretical framework

We conducted a qualitative study with medical students following the traditional or integrated curriculum. IPE and teamworking are integral to the delivery of high quality healthcare and patient safety (WHO 2010, 2011). Therefore, medical students’ perceptions and experiences of working with nurses are subject to the number of interprofessional collaboration opportunities offered during their training. This is consistent with the world view that reality is subjective, socially constructed and shaped by a person in line with their experiences that is manifest in subjectivist epistemology and constructivist ontology within the interpretivist paradigm (Guba and Lincoln 1994; Bryman and Bell 2011). Subjectivist epistemology and constructivist ontology lend themselves to qualitative research with an emic stance and a focus on gathering rich data to inductively generate theory (Kaya 2013; Bunniss and Kelly 2010). Consequently, we undertook this qualitative study within the interpretivist paradigm informed by subjectivist epistemology and constructivist ontology.

Curricula which are designed to facilitate interprofessional collaboration and patient safety must to take into account the distinct level of competence that is expected of each healthcare related discipline (WHO 2010, 2011). However, competence in relation to any discipline can be conceptualised in different ways depending on the epistemological, ontological and philosophical position that is adopted (Lum 2009). Data collection and analysis in this study was informed by a theoretical framework which integrated pertinent elements of the General Medical Council’s (GMC) (2015) standards for medical education relating to interprofessional education, and Cheetham and Chivers’ (1999) conceptual framework of professional competence (see Fig. 2).

### Setting

**Fig. 1:**
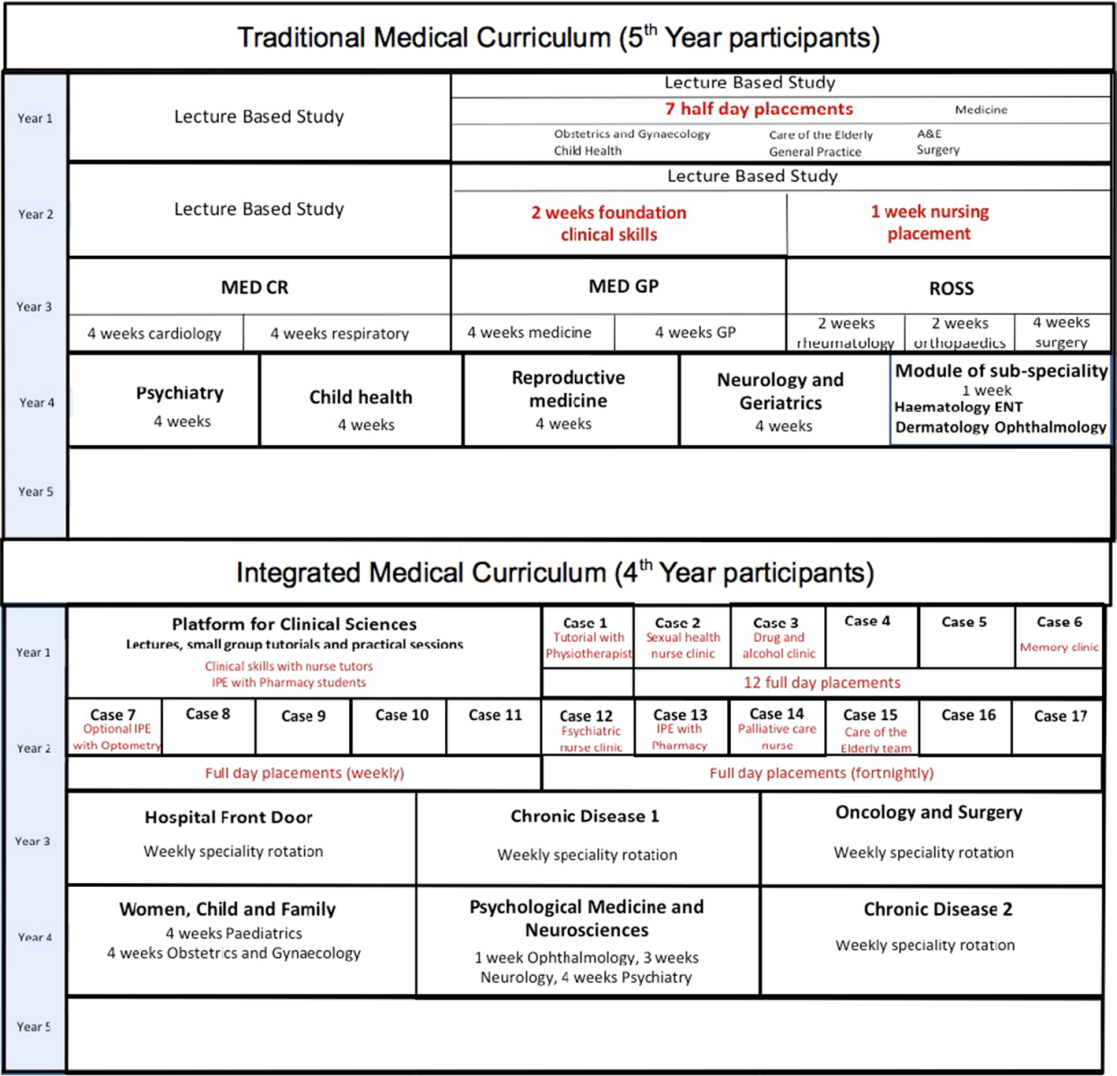
Overview of the medical curricula years 1–4

In 2013, the Russell Group University medical school in this study underwent curriculum reform in which the opportunities to learn from and about nurses in practice and academic settings were replaced by an integrated curriculum which provided new opportunities for medical students to learn from students and professionals from a more diverse range of healthcare related disciplines (see Fig. 1). In the traditional curriculum, medical students were provided with specific opportunities to learn from with and about nurses at set periods during the first and second years of training. In the first year on the traditional curriculum, students had seven half-day clinical placements in which they had the opportunity to interact with nurses. In the second year, medical students were taught foundation clinical skills over a 2-year period by medical and nursing tutors. Medical students in the traditional curriculum also had 1-week nursing placement during which they shadowed a nurse in clinical practice which provided them with an opportunity to learn about what nurses do and how they contribute to patient care.

The new integrated curriculum replaced the clinical and taught sessions with nurses with a more structured approach to IPE, involving students from different healthcare professions learning with, from and about each-other (Barr 2014). In the integrated curriculum, medical students learn about clinical sciences alongside pharmacy students in lectures, practical sessions and small group tutorials. The integrated curriculum also sought to foster IPE through structured tutorials (some delivered by nurse tutors) in the first and second year as well as 1-day clinical placements with a variety of healthcare professionals in the first and second year (see Fig. 1). However, it is unknown what impact the curriculum change in this university has had on medical students’ perceptions of their interactions with nurses.

### Data collection

Data were gathered through individual semi-structured interviews undertaken with a qualitative narrative approach (Riessman 2008) by a member of the research team (EL). The narrative interview schedule was piloted for face and content validity before being used to gather data in interviews where participants were invited to recall experiences of working with nurses. The initial outline interview schedule which sets out the key stem questions is shown in Table 1. However, the interview schedule evolved during the course of the study, as we identified new insights into the participants’ perceptions of their interactions and team-working with nurses on the traditional and integrated curricula. Questions relating to novel insights identified in individual participants’ experiences were incorporated into the interview schedule for subsequent interviews. Additionally, we used a narrative inquiry approach (Mitchell and Egudo 2003; Wengraf 2004; Spector-Mersel 2010) in the interviews which allowed insight into participants’ perceptions and how they made sense of the situations they encountered. Exploring perceptions in relation to interprofessional relationships was essential in this study because they can directly affect attitudes, interactions and the quality of healthcare (GMC 2016; Hall et al. 2016).

**Table 1:** Initial outline interview schedule

The participants in this study will be asked the following stem questions in the individual interviews. Additional questions may be asked in order to further clarify specific points that the participants make. Please note that these stem questions changed as the study progressed and new insights were identified in relation to the participants perceptions of their interactions and team-working with nurses on the traditional and integrated curricula
Please tell me a bit about yourself
What is your year of study?
Which curriculum have you engaged with (C21 or traditional)?
What role have nurses played in your academic training so far?
Thinking back on your time as a medical student so far, could you tell me about the most memorable interaction you have had with a nurse? (Can be positive or negative)
How did you feel during the event?
Did you ever talk to anyone about the support you received?
How do you feel this interaction/experience has affected your interaction with other healthcare professionals?
Before this interaction/experience, what was your understanding of the role of a nurse?
Were you aware of the support they can provide?
Do you think your perceptions have changed?
What impact do you think curriculum has had on your perceptions of your interactions with nurses?
When you have been in clinical practice, what have your medical colleagues told you about nurses?
How do you think junior doctors perceive nurses? Have you any examples of doctors’ interactions with nurses?
What do you think medical students should know about the role of nurses in clinical practice? Why?
Is there anything else that you would like to add about the role nurses play in supporting medical students?

The participants were medical students recruited via email from two separate cohorts following differing curricula within a UK Russell Group University. Purposive sampling (Palinkas et al. 2015) was used to recruit an equal number of male and female medical students in the fourth (n = 6) or fifth-year of their study (n = 6) engaging in the integrated and traditional curricula respectively. Gender can influence perceptions (Van Wyk et al. 2016), so we also sought to achieve equal gender representation amongst the participants. Participants were recruited until data saturation was achieved for the key concepts in this study. All of the participants took part in a one-off individual interview. Ten participants were interviewed face to face in a quiet room away from the medical school, one participant was interviewed on the telephone and one participant was interviewed via Skype.

### Data analysis

**Fig. 2:**
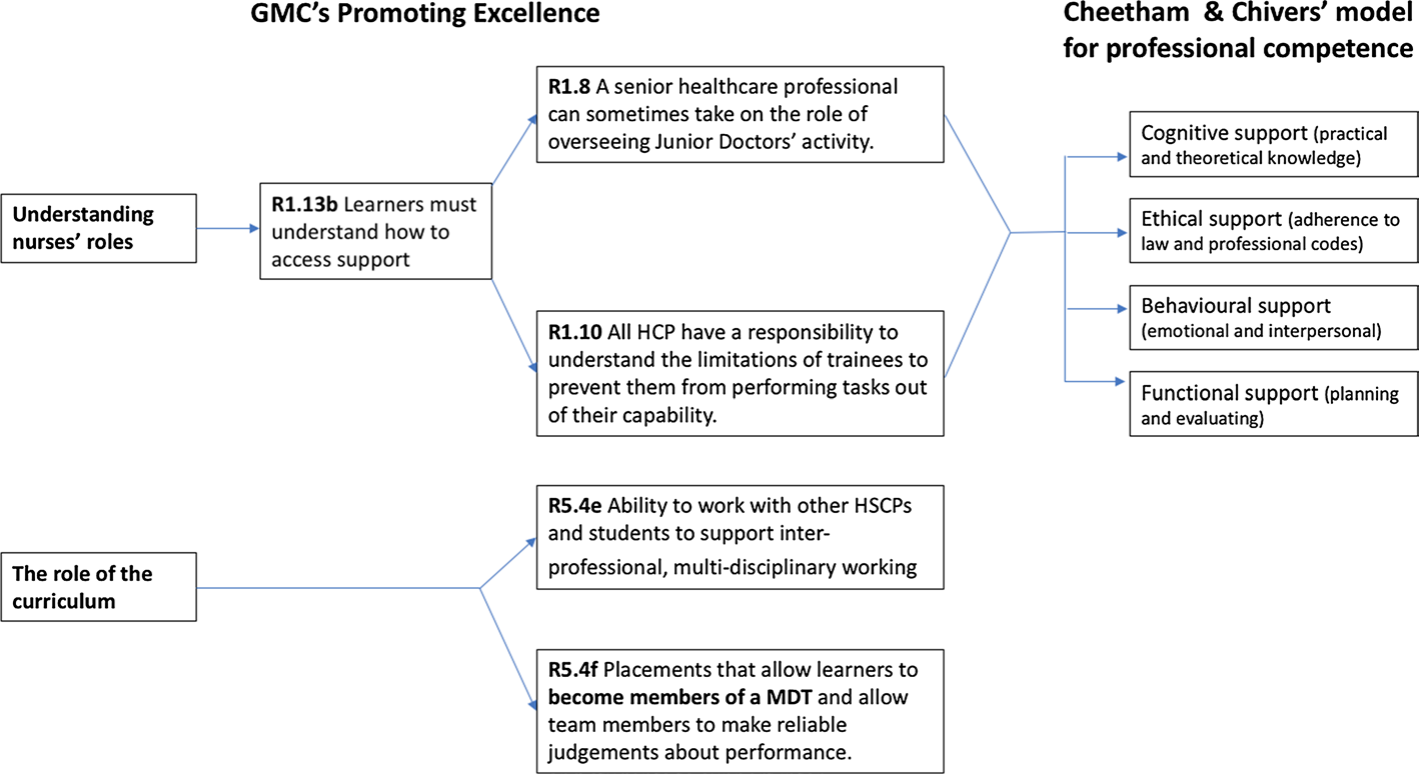
Theoretical framework

**Fig. 3:**
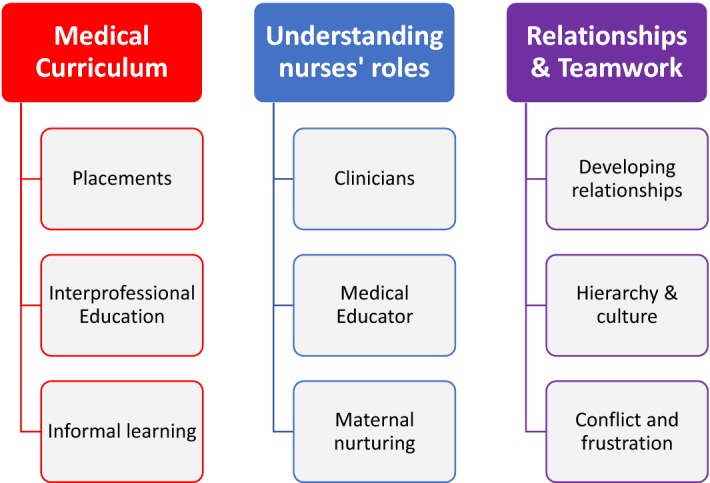
An overview of themes and subthemes

The interviews, which varied in duration from 10 to 30 min, were digitally audio-recorded and transcribed verbatim. Narrative data analysis was undertaken using NVivo 11. Data analysis was initially undertaken using an a priori theoretical framework based on the GMC (2015) standards for medical education and Cheetham and Chivers’ (1999) framework for professional competence (see Fig. 2). The coding framework was further developed in an inductive iterative process around identification of themes as the study progressed. Initial data analysis was undertaken by EL and independently reviewed by each of the other researchers (RS, KW and AB). All researchers met to discuss the application of the coding framework and data analysis and a consensus was reached on the main themes and subthemes (see Fig. 3).

### Ethics and consent to participate

This study was granted research governance and ethical approval by the Cardiff University Medical School Medical Research Ethics and Conduct panel in December 2016. All of the participants gave their written informed consent to take part in this study.

## Results

There were no major differences in the accounts of the participants who undertook the traditional and integrated curricula in relation to their perceptions of their interactions and team-working with nurses. We identified three main themes that were congruent with our theoretical framework, i.e.: medical curriculum, understanding nurses’ roles as well as relationships and teamwork. The themes and subthemes (Fig. 3) are illustrated by a selection of quotations is presented which epitomise the sentiments expressed by the participants in this study. We have also highlighted instances in which the participants expressed contrasting views in relation to each theme. The participant demographics are set out in Table 2 with the use of pseudonyms to maintain confidentiality.

**Table 2:** An overview of participant characteristics

Pseudonym	Gender	Year of study	Curriculum
Ella	Female	4	New, integrated curriculum
Isla	Female	4	New, integrated curriculum
Sophia	Female	4	New, integrated curriculum
George	Male	4	New, integrated curriculum
Noah	Male	4	New, integrated curriculum
Oscar	Male	4	New, integrated curriculum
Ava	Female	5	Former, traditional curriculum
Mary	Female	5	Former, traditional curriculum
Olivia	Female	5	Former, traditional curriculum
James	Male	5	Former, traditional curriculum
John	Male	5	Former, traditional curriculum
Tom	Male	5	Former, traditional curriculum

### Medical curriculum

All participants asserted that their perceptions of nurses had been influenced by interactions within the medical curriculum. Participants said that the medical curriculum had increased their awareness of the variety of nursing roles and responsibilities, which in turn enabled them to challenge pre-existing stereotypes. The fifth-year participants drew particular attention to the nursing placement within the traditional medical curriculum. It had enabled them to learn more about the different nursing roles and altered their perceptions:

“We underwent a nursing placement for a week and that gave us a bit more insight into their roles and their responsibilities.” James (Y5M)

Fourth-year participants described the removal of the nursing placement from the integrated curriculum as a lost opportunity to develop medical students understanding of nurses’ roles. However, fourth-year participants on the integrated curriculum reported having earlier clinical exposure than those on the traditional curriculum, which provided them with valuable experience of interacting with other healthcare professionals.

The participants’ narratives also suggested that the length of clinical placement has an influence on medical students’ perceptions of nurses. Many participants on both curricula pathways suggested that longer placements such as those on the traditional curriculum fostered better interprofessional relationships and team-working because nurses and medical students had enough time to get to know each other:

“I think we would be more aware of what they do and also do much more, if we were able to build up a relationship which we’re not currently able to because we’re not there for long enough.” Sophia (Y4F)

A participant on the integrated curriculum stated that their experience of numerous short clinical placements had exposed them to working with a variety of nurses which facilitated the development of a greater understanding of the varied roles of nurses. This participant opined that varied shorter clinical placements in the integrated curriculum were ideal because they enabled students to get a wider view of all the roles that nurses can play instead of what they do in one particular setting:

“I’ve seen nurses in all different specialities so I’ve kind of got to know the nurse population as a whole, rather than just one team of nurses.” George (Y4M)

Every participant stated that teaching sessions with student nurses were beneficial for medical students as they enabled them to develop a better understanding of the role of a nurse. The participants expressed disappointment that there were minimal teaching sessions with nursing students in both curricula as it was felt that a lack of interactions with nursing students was a missed opportunity for IPE:

“We don’t really interact much with student nurses, which is a shame because you spend most of your career interacting with nurses.” Noah (Y4M)

### Understanding nurses’ roles

There was a unanimous view amongst the participants that nurses played a key role in patient care regardless of the curriculum. Some participants described the role of the nurse in relation to fundamental aspects of care such as washing and feeding patients, which are synonymous with the role of healthcare support workers. All participants reported that they found the different types, grades and roles of nurses in clinical practice confusing. Participants felt that their lack of understanding of nurses’ different grades could lead to hierarchical attitudes, poor relationships and ineffective teamworking. The consensus amongst the participants was that they could work more productively with nurses if they had a more comprehensive understanding of nurses’ roles:

“If you want to be able to interact best with people to make the care of patients as seamless as possible, I think it’s important to know whose job is which, whether it’s the nurse, healthcare professional, specialist nurse.” John (Y5M)

The participants also indicated that they recognised the role of nurses as medical educators who supported them to develop cognitive, personal, functional and ethical professional competence especially in clinical practice. Nurses were reported to support medical students on the traditional and integrated curriculum to develop their knowledge and procedural ability in clinical practice. The participants maintained that nurses’ expertise provided medical students with unique insights into the delivery of medical care to patients in clinical practice. Senior nurses were cited by students on both curricula as possessing a vast knowledge base and the ability to provide in-depth clinical insights to medical students:

“She (the nurse) was really helpful in teaching me how to take a history and everything, which I hadn’t expected from a nurse. I didn’t realise they had such a key role.” George (Y4M)

However, one participant pointed out that even though nurses were recognised as medical educators they were not always willing or able to fulfil some aspects of the role of a valid supervisor for medical students:

“Sometimes, I‘ve done a skill and they [the nurses] are like, ‘oh, I can’t sign that off, I don’t know what it is.’” Oscar (Y4M)

It must be acknowledged that in the new integrated curriculum, nurse tutors are involved in teaching clinical skills which may have given rise to a misconception about the support that other nurses can or should provide with regards to signing off clinical skills in clinical practice. This is a subtle but important point as nurses in clinical practice were unaware of the changes to the curriculum at the time of the study and would previously come across medical students on the traditional curriculum who would have already had a nursing placement and arguably, a better insight into the role of nurses. Therefore, the encounter reported by this participant may have been a case where the changes to the curriculum influenced the nurse’s willingness or confidence in their ability to undertake the role of a medical educator in relation to signing of clinical skills.

All participants maintained that nurses helped medical students to develop their knowledge and confidence during clinical placements. Even though there are male nurses, the participants also recounted their experiences of nurses playing a comforting, maternal role in their interactions with medical students. All participants also recalled instances when they had seen nurses providing guidance to junior doctors after making mistakes and nurses providing junior doctors with “maternal comfort” during emotionally challenging situations:

“We all really appreciate and admire her (the nurse) and respect her knowledge source. She’s a very calming, motherly influence.” Isla (Y4F)

### Relationships and teamwork

The participants’ accounts suggested that there are a number of different factors which influenced medical students’ relationships and team-working with nurses. On one hand, the participants asserted that nurses who played the role of medical educator were generally more approachable and accommodating than their medical counterparts. As a result, some participants on both curricula said that they often chose to report to nurses when they began working on a new ward because nurses knew how the ward works and are willing to help:

“They’re often the people you first approach because they’re quite accommodating a lot of the time, and they know what’s going on more than most of the doctors.” Sophia (Y4F)

Other participants spoke about finding it intimidating to ask for help from nurses even if they were approachable which resulted in limited engagement in team-working with nurses and other aspects of patient care:

“You don’t really want to ask them if you can do stuff. It can lead you to, sort of, hiding away and not get involved as much.” Ella (Y4F)

All participants maintained that nurses helped medical students to develop their knowledge and confidence during clinical placements and they were mindful of showing nurses due respect to facilitate good working relationships which thus enabled them to receive teaching and support from nurses:

“If you don’t respect them, they’re not going to respect you and they’re not going to help you out.” Ava (Y5F)

Many participants on both curricula spoke about how junior doctors advised them of the importance of fostering positive working relationships with nurses to facilitate more effective and enjoyable interprofessional work and the dangers of failing treating nurses badly:

“Treat the nurses well and don’t get on their bad side because they will be horrible to you.” Tom (Y5M)

Although we did not ask any questions about gender in this study, the participants referred to the impact that gender had on their ability to develop working relationships with nurses. There was an underlying assumption in the participants accounts that all nurses are female even though this is not the case. The vast majority of participants described a perceived difference in nurses’ attitudes towards male and female medical students irrespective of curricula. Nursed were reported to have a less positive demeanour towards women in medicine than towards male in medicine. For example, the nurses would only learn the names of male doctors:

“It happens all the time… my female colleague said that all the nurses knew all the male doctors’ names on the ward.” Oscar (Y4M)

The participants reported that senior doctors warn female medical students that tension could arise in their future relationships with nurses as a result of their gender but this was something that the female students on both curricula had not experienced:

“A male doctor said to me: you’ll have trouble because nurses don’t like female medical students. But I haven’t found that to be true from my personal experience.” Ava (Y5F)

## Discussion

Our study sought to establish the impact of curriculum reform on medical students’ perceptions of their interactions and team-working with nurses. We did not find any notable differences in the accounts of participants on the old traditional curriculum and the new integrated curriculum about their working relationships with nurses. The medical students in this study valued their relationships with nurses. They recognised how nurses could support their educational needs as students and their future roles as junior doctors in providing safe and effective patient care. The participants on both curricula highlighted some barriers to team-working such as a poor understanding of nurses’ educational and professional roles, mistaken views about doctors and nurses in the professional hierarchies in healthcare, and stereotypical views about the relative knowledge and abilities of nurses and doctors.

The integrated curriculum used in the medical school involved in this study was said to have delivered some positive outcomes, with early clinical exposure reported to be beneficial for developing interprofessional relationships. However, the participants also felt that changes made in the integrated curriculum had resulted in lost opportunities for fostering good interprofessional teamwork with nurses due to the removal of a nursing placement. A lack of IPE with nurses and shorter clinical placements were said to have an impact on medical students’ interactions and perceptions of nurses. Educational curricula that foster meaningful social relationships in which students and educators from different professions learn from with and about each other have been shown to foster interprofessional teamwork (Ateah et al. 2011; Hean et al. 2012; Reeves et al. 2016). Facilitating the development of meaningful interprofessional relationships with the curriculum is important because it challenges any gaps or misconceptions in a person’s knowledge, attitude or perception of their own discipline in relations to other professions which facilitates effective teamwork (Ateah et al. 2011; Reeves et al. 2016). Therefore, our study findings suggest that there is work to be done to embed more opportunities within the curriculum for medical students to develop productive relationships with nurses that enable them to work effectively with nurses in clinical practice.

There was a unanimous view amongst participants that nurses played a key role in patient care by delivering fundamental hands on patient care. All the participants also highlighted that nurses were medical educators who played a supportive nurturing role in the professional development of medical students and junior doctors. Participants reported that their ability to work productively with nurses was hindered at times by a lack of knowledge about the expertise of nurses of different professional grades who they encountered in clinical practice. Some participants seemed to be unaware of the role nurses play in performing clinical skills, prescribing and leading clinics. The participants accounts point to limited opportunities with both curricula to learn about the ability of nurses to undertake some aspects of medical work in order to improve patient care, which have a direct impact on interprofessional relationships between doctors and nurses (Williamson et al. 2013; Powell and Davies 2012). The participants accounts about the impact of a lack of insight into the wider contribution of nurses to patient care is supported by other reports (Salhani and Coulter 2009; Allen 2015) of blurred professional boundaries between doctors and nurses causing interprofessional tension, conflict and poor team-working. There is also a contrasting view held by some (Powell and Davies 2012) that blurred professional boundaries are a boon for interprofessional working because they foster more egalitarian interactions in absence of hierarchy. However, the latter view is not supported by the accounts of participants on both curricula about their interactions and team-working with nurses. Instead the evidence from this study points to some considerations that need to be taken into account with regards to curriculum design which fosters IPE and team work.

Our findings suggest that there are some aspects of the new integrated curriculum which need to be revised to create more opportunities for medical students to learn about the role of nurses. This is important as it is widely accepted (Hammick et al. 2007; Ateah et al. 2011; Reeves et al. 2016) that curricula which foster interprofessional teamwork take into account the specific learning needs of students and enable them to understand the role of other healthcare professionals in the high-quality patient centred care. The importance of role understanding as a key facet of interprofessional teamwork is also highlighted in a diverse range of policy and practice drivers (WHO 2010; GMC 2016). A better understanding by medical students of the expertise of nurses in relation to medical practice is important as uncertainty about professional roles which blurs professional boundaries can allow misconception and stereotypes to develop about colleagues from other professions (Fiordelli et al. 2014). Challenging professional stereotypes and improving interprofessional working requires a shared understanding of the roles of different professions, as well as a consensus on the delegation of and responsibility for different elements of work (Powell and Davies 2012; Salhani and Coulter 2009).

Participants were aware of how nurses’ support could help them develop their cognitive competencies, such as knowledge acquisition and procedural techniques. This finding is congruent with Samuriwo et al.’s (2016) unpublished study in which nurses said that they supported junior doctors to develop their capability in relation to key elements of their cognitive, functional and ethical competence in medical practice at the onset of their clinical careers. An awareness of the support nurses can provide in the development of professional competence has added importance during the transition from medical student to junior doctor when support from more senior doctors is often lacking (Brennan et al. 2010; Sturman et al. 2017). The participants recognised that medical students’ and junior doctors’ understanding of nurses’ role in supporting them to develop their functional, behavioural and ethical competence could be further developed to ease this transition.

Although some participants perceived nurses as a comforting figure within the hospital, most failed to acknowledge the support that nurses can offer in the development of emotional competence. Newly qualified junior doctors are reported to experience high levels of burnout (Sturman et al. 2017; Teunissen and Westerman 2011), so it is important that they are aware of and can access the emotional support that colleagues, such as nurses, can provide (Petri 2010). However, it should be noted that the responsibility to provide interprofessional support lies with all healthcare professionals and is not solely the remit of nurses (WHO 2010, 2011).

Our findings indicate that some medical students view nurses’ roles as akin to the healthcare support worker are congruent with Hean et al.’s (2006) assertions about established stereotypes more than a decade before. Hean et al. (2006) asserted that medical students entering university can have established, stereotypical ideas of doctor and nurse characteristics. Historically, nurses seldom influenced patient management as they were perceived to be inferior and subservient to doctors (Fagin and Garelick 2004). These hierarchical views about the doctor–nurse relationship have evolved as nursing has become increasingly professionalised (Fagin and Garelick 2004). However, ideas relating to the stereotype of the caring, subordinate nurse are evident in our findings, as some participants referred to nurses as a maternal figure and did not recognise that nurses also had a position of authority. Professional stereotypes about doctors and nurses can give rise to attitudes and behaviour which can create interprofessional conflict which compromises patient safety (Braithwaite et al. 2013; Powell and Davies 2012). Professional stereotypes and hierarchies are a social construct within the clinical environment that must be challenged in order to establish effective interprofessional collaboration (Ateah et al. 2011). Therefore, our findings suggest that more needs to be done within the curriculum to ensure that medical students have a better understanding of the role of nurses and how they contribute to patient safety in the contemporary context.

The participants in this study also felt that gender had an impact on the nature of their professional relationship with nurses who were said to be more favourably disposed towards male students in comparison to the female students which could give rise to tension. Evidence from studies in different settings (Wear and Keck-McNulty 2004; Van Wyk et al. 2016), suggests female nurses in particular are said to demonstrate greater hostility towards their female medical colleagues. Zelek and Phillips (2003) opined that that hostility arose from the diminished power imbalance when both doctors and nurses were female. Gender is said to be the main contributor to perceived power within the doctor–nurse relationship, so when both doctors and nurses are female, attitudes are more egalitarian (Zelek and Phillips 2003). Egalitarian relationships may allow nurses to feel more confident in approaching female doctors, but they may also result in nurses showing greater hostility towards female doctors (Zelek and Phillips 2003). It is important to note that many of these findings and opinions are based on the assumption that all nurses are female and there is little or no consideration of the nature of male nurse-female doctor relationship in wider literature.

The participants identified ways in which positive relationships could be fostered through IPE with nursing students, nursing placements and opportunity to be integrated into multi-disciplinary teams. Even though there is evidence which highlights the effectiveness of the educational interventions suggested by participants, none of these measures or interventions were reported to have been implemented within the new, integrated medical curriculum. IPE may be logistically difficult to implement, but it tends to be well received by students and helps students to develop the skills necessary for collaborative practice (Reeves et al. 2016; Darlow et al. 2018). Participants from the integrated curriculum recalled engaging in IPE sessions with pharmacy and optometry students only. Given that doctors and nurses work closely within the multi-disciplinary team, perhaps IPE with nursing students should also be implemented. However, Hammick et al.(2007) assert that the ability of IPE to change perceptions is open to question, and medical educators need to be mindful that IPE may worsen hierarchical attitudes relating to doctors and nurses. Conversely, a more recent systematic review (Reeves et al. 2016) suggests that IPE improves attitudes relating to medical practice.

Nursing placements for medical students have also been shown to challenge professional stereotypes (Helmich et al. 2010). There is a limited body of literature on the use of a nursing placement but Helmich et al. (2010) report that medical students’ perceptions about nursing roles are challenged during nursing placements, which develops their overall understanding and appreciation of nurses’ contribution to medical education and patient care. Participants that engaged in the integrated curriculum stated that a positive outcome from the medical curriculum change was the early clinical exposure. A systematic review (Dornan et al. 2006) found that early clinical exposure can help students learn about the structure of the clinical environment and about the roles of other healthcare professionals. Participants reported that they were given opportunities to interact with a wide variety of nurses during the short clinical placements, which increased their understanding about the various nursing roles. Shorter clinical placements within the integrated curriculum (1-week as opposed to 4-week rotations) may allow medical students to learn about the differing nursing roles, but it is not clear if they are of sufficient duration to enable medical students to work alongside nurses as part of healthcare team.

The participants recalled experiencing the dynamic of interprofessional teamworking but said that they did not feel a legitimate *part* of the team during their short clinical encounters. The short clinical placements were reported to limit the opportunities for developing strong, interprofessional relationships which meant that medical students could only interact transiently with nursing colleagues. This is echoed in Milne et al.’s (2015) ethnographic study which found that short placements prevent the development of meaningful relationships between junior doctors and nurses. These transient interactions appeared to shape the participants’ perceptions of nurses. Further research is needed to ascertain how best to provide medical students with a diverse range of appropriate role models from nursing and other healthcare professions who support them to develop their competence and deliver high quality patient care in clinical practice.

### Strengths and limitations

To the best of our knowledge, this is the first study to have explored how changes to the medical curricula have impacted on medical students’ interactions with nurses. Some novel, interesting insights have been identified but our study is not without its limitations. Firstly, only medical students were interviewed and so the views expressed can only be interpreted from the medical students’ perspective. Secondly, time constraints limited further exploration of a contrasting view relating to the effect of gender on doctor–nurse relationships. This study only involved 12 medical students from one medical school which limits the transferability of the findings to other settings. However, our findings are congruent with and build on wider literature about the support that nurses provide to medical students and juniors doctors.

In particular our findings are congruent with the notion expressed in a previous unpublished study (Samuriwo et al. 2016) that the support which nurses provide to foster the development of professional competence in medical practice, is in effect a part of the invisible work of nurses that contributes to the hidden curriculum of medical education. The concept of the invisible work that nurses do in relation to the organisation of medical work has also been articulated in a theory by Allen (2015), which is predicated on a range of studies that have examined the organisation of labour in different healthcare settings.

Given additional time, a mixed methods approach could have been adopted to improve the applicability of the findings. The transferability of our findings is enhanced by the purposive sampling of participants, which resulted in an equal gender split and representation from two different medical curricula. It must also be acknowledged that participants involved had completed varying amounts of their medical training which could have affected their interactions and perceptions of nurses. However, given the curriculum change, we judged it appropriate to collect data at these different time points.

It is also possible that participants may have given what they believed to be socially desirable responses. Efforts were made to limit this by use of a narrative approach with questioning that focused on the participants’ memorable interactions with nurses (Riessman 2008). Additionally, data were gathered by a medical student (EL) which reduced the social distance between researcher and participants. Whilst this acted to reduce social desirability bias, it was necessary to maintain professionalism and a degree of formality and so, a balance between good rapport and task-orientation was established. Reporting bias was minimised through the independent review and agreement of data coding and analysis by the research team. The rigor and credibility of this study were further enhanced by a literature review that focused on findings and key concepts identified in this study. This also acted as a form of triangulation that highlighted knowledge gaps (Farmer et al. 2006).

## Conclusion

This qualitative study gathered data though semi-structured, narrative interviews with medical students from two different curricula. The aim of this study was to ascertain the impact of medical curriculum reform on medical students’ perceptions of their interactions and teamworking with nurses. We found that medical students value nurses’ input in medical education, but there appear to be limited opportunities for medical students to develop meaningful relationships with nurses that foster interprofessional team-work and support the development of professional competence in medical practice. Medical educators need to be mindful of the constellation of support that nurses can provide to medical students to develop their professional competence in the design and delivery of medical education and training. Medical curricula can be further improved through the incorporation of more structured opportunities for medical students to learn from and work with nurses, especially in clinical practice. Further research is needed to establish how best to embed more opportunities for IPE between medical students and nurses.

## Abbreviations

IPE: Interprofessional education
GMC: General medical council
